# The Role of Social Media in an Inspirational Approach to Product Design and Designer Performance

**DOI:** 10.3389/fpsyg.2021.729429

**Published:** 2021-09-09

**Authors:** Man Gao, Nazik Hangeldiyeva, Mahri Hangeldiyeva, Fahad Asmi

**Affiliations:** ^1^Anhui Institute of Public Security Education, Hefei, China; ^2^School of Art and Design, Zhejiang Sci-Tech University, Hangzhou, China; ^3^School of Economics and Management, Zhejiang Sci-Tech University, Hangzhou, China; ^4^Department of Science and Technology of Communication, University of Science and Technology of China, Hefei, China; ^5^Key Laboratory of Immersive Media Technology (Anhui Xinhua Media Co., Ltd.), Ministry of Culture and Tourism, Hefei, China

**Keywords:** novelty-focused product design, efficiency-focused product design, designer performance, enterprise social media, public social media

## Abstract

Social media has encouraged a large number of organizations to design their work patterns to facilitate their employees through social media adoption. This study examines the effects of enterprise social media and public social media on the novelty-focused product design, and efficiency-focused product design that eventually explain the performance of the designers. Empirical analysis based on time-lagged, multi-source data set indicate that both enterprise social media and public social media are an important source of product designer's inspiration which are positively related to novelty-focused design and efficiency-focused product design. Results further indicate that efficiency-focused product design has a stronger impact on designer performance than novelty-focused design. Theoretical implications and practical implications are discussed in the later sections.

## Introduction

Over the past two decades, an increasing number of organizations are adopting social media to support creativity and innovation to enhance product performance (Vuori et al., 2012; Bharati et al., 2015; Myers, 2015). Social media is playing a critical role in supporting innovation-driven organizational strategies, therefore, recent studies are investigating how social media platforms have affected product and service performance (Li et al., 2018; Islam et al., 2021). However, for social media to affect product performance through innovation requires individuals to differentiate their intentions in product designing. For example, social media is a dominant contributor to designing efficient and innovative products to satisfy consumer needs (Rathore et al., 2016). A large amount is invested by the companies to leverage social media to support its employees. Expecting to realize efficient and innovative product designs that can help gain market share. However, the expected value of a company's investment in social media is often not realized (Cao and Ali, 2018; Yu et al., 2018), creating a challenge for the firm to adopt appropriate strategies to support employees in creating novel products.

Despite the fact that many researchers have looked at the impact of social media on internal organization interaction among individuals, and teams (Sherf and Venkataramani, 2015; Hu et al., 2018; Bao et al., 2020; Liang et al., 2020), and external interaction with customers (Islam et al., 2020; Zafar et al., 2021), we still have limited knowledge of the role of social media. Specifically, there is limited empirical research that provided information on how the use of social media affects product design (D'Andrea et al., 2015). Product design can be categorized into novelty-focused product design (NFPD) and efficiency-focused product design (EFPD) (Gerwin and Barrowman, 2002; Micheli and Gemser, 2016). The two product designs are not mutually exclusive. Rather, both can be part of any product design at the same time. However, due to the designer's unique design orientations, both NFPD and EFPD are critical sources of product success. Although, organizations and individuals use social media to help communicate with co-workers, and customers to improve product design (D'Andrea et al., 2015; Do, 2016; Rathore et al., 2016), yet, how social media use affect NFPD and EFPD remains a challenge.

In organizations, employees not only use enterprise social media platforms, but also use public social media platforms to interact with potential customers (Ali et al., 2019a; Wei et al., 2020), and gain resources to facilitate their product design. Social media is used by employees for their work-related purposes and social related purposes simultaneously (Cao and Yu, 2019; Liang et al., 2020). Besides, studies have suggested that employees use both enterprise social media, as well as public social media platforms to satisfy their needs (Cao et al., 2012, 2016; Laitinen and Sivunen, 2020). Accordingly, employees use enterprise social media, and public social media to satisfy their needs. However, enterprise social media alone cannot always satisfy their work-related needs, for instance, organizations often face budgetary challenges to provide extended social media facilities. In addition, potential customers and professionals having useful knowledge may reside outside the enterprise social media networks. Thus, public social media platforms are considered a useful resource-generating facility for employees engaged in product designing (Gregg, 2010; Yang et al., 2021). Therefore, we contribute to the research on social media and extend the literature by investigating the simultaneous role of enterprise social media and public social media to support NFPD and EFPD.

This study builds on organizational learning (OL) theory (Real et al., 2006) to investigate the link between two types of social media platforms used by employees and product design, which eventually explain designer performance. The OL theory states that different practices that enable performance rely on learning practices. Thus, we suggest that the development of NFPD and EFPD will need unique social media capabilities. Since, enterprise social media, and public social media enable employees to interact, communicate, exchange ideas, share knowledge, and understand potential customers (Choi, 2019; Ali et al., 2020a; Cao et al., 2020; Laitinen and Sivunen, 2020), we argue that two types of social media platforms could support NFPD and EFPD, which consequently enhance the performance of the designing employee.

This research adds to the existing body of knowledge in at least three ways. First, this research adds to the body of knowledge about social media by explaining the complex linkage between social media use, product design. Second, we investigate the link between a particular social media platform (enterprise social media and public social media) with the product design focus of the employees (NFPD and EFPD). Third, our study theoretically based on the OL theory highlights the role of social media platforms on design employee's performance through NFPD and EFPD. Thus, this study provides novel insights into how different focus of designers can explain their value and contributions for the organizations.

## Theoretical Background and Hypotheses

### Organizational Learning Theory

OL theory suggests that learning provides a foundation to develop distinctive capabilities, which eventually enable higher performance outcomes (Real et al., 2006). More precisely, it indicates that learning is a dynamic process that includes acquiring, sharing, transforming, and applying knowledge in a way that facilitates improved services and products (Kane et al., 2014). OL theory further suggests that knowledge exploration and knowledge exploitation are two main components of the learning process through which employees acquire, exchange, develop, and use knowledge where needed (Kane and Alavi, 2007).

**Figure 1:**
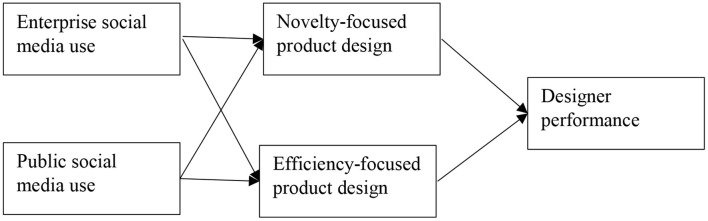
Conceptual model of the study.

The literature on knowledge exploration and knowledge exploitation recognizes the use of information technology and suggests that information technology such as social media has important implications for knowledge management processes and learning activities (Alavi and Leidner, 2001; Benitez et al., 2017; Ali et al., 2020b). More recently, researchers have discovered that using social media improves knowledge management, creativity, and performance (Davison et al., 2018; Khan et al., 2019; Wei et al., 2020). For instance, Cao et al. (2020) noted that social media use facilitates team knowledge creation and performance. Ali et al. (2020a) found that social media facilitates knowledge exploration and exploitation of team members, leading to improved innovative solutions. Thus, this study in line with previous literature suggests that social media use may positively facilitate designers in developing NFPD and EFPD. Particularly, we propose that enterprise social media and public social media, both are related to NFPD and EFPD by designing employees. The study's conceptual model is shown in Figure 1.

The literature on product design has suggested that product design is a social process in which different actors (i.e., employees, customers, and distributors) are involved. The involvement contributes to a product design that is novel and efficient to satisfy user needs. Involvement of potential customers, for example, may enable designers to come up with new features and fixtures, enhanced product quality and size, renewal of existing product design according to the needs of the customers, and understanding the durability and creative design needs of the end-users. D'Andrea et al. (2015) noted that using social media to engage customers enables better product design and features that increase product performance (Yang et al., 2019). Therefore, enterprise social media, and public social media both are important technological platforms that contribute to product design that is novel and efficient, that eventually explains the performance of the employees designing the product.

### Social Media Use and Product Design

Enterprise social media use as a technological tool enables interaction, communication, and knowledge exchange among organizational members may help product design (Laitinen and Sivunen, 2020; Ma et al., 2020; Wei et al., 2020). We propose that enterprise social media use supports NFPD and EFPD. Firstly, enterprise social media use facilitates designers to access diverse knowledge from organizational members for improving design novelty in product designing (Ali et al., 2020b). It provides more resources, ideas, and content that helps designers re-think the product with novel insights (Wagner et al., 2014). Accordingly, designers can generate novel ideas in designing and producing products. For example, Ali et al. (2020a) found that the use of enterprise social media enables employees to acquire knowledge resources and integrate those resources to generate novel ideas for products and services. Accordingly, we propose that the use of enterprise social media enables NFPD development.

Secondly, enterprise social media can also facilitate EFPD. For instance, discussion with colleagues from other departments, such as sales and marketing, manufacturing gives access to alternative knowledge, and market insights (Moqbel and Fui-Hoon Nah, 2017; Aboelmaged, 2018; Ali et al., 2021), which lends new perspectives to understand existing product designs. Thus, employees engaged in designing products can reevaluate existing product design, eliminate defects, and increase the efficiency in their product design, leading to increase product use and reducing manufacturing cost, while enhancing user satisfaction. In this way, designers can simplify product design, and transform design according to the requirements of the manufacturing, sales, and marketing departments who have close contact with customers. Thus, we propose that the use of enterprise social media will facilitate designers in a way that enables them to generate EFPD. In summary, we develop the following hypotheses.

H1: Enterprise social media use is positively associated with novelty-focused product design.
H2: Enterprise social media use is positively associated with efficiency-focused product design.

Similarly, we argue that public social media platforms are enablers of exploration and exploitation of knowledge leading to facilitate designers in developing innovative and efficient product designs. Firstly, enterprise social media extends knowledge exploration by increasing interaction with external resources of knowledge and ideas (potential customers) (Cao et al., 2020; Naeem and Ozuem, 2021). The utilization of public social media platforms improves the knowledge acquisition from external knowledge stocks to the organization, providing an opportunity to integrate with internal knowledge to generate innovative ideas that could satisfy customer expectations (Peppler et al., 2011). Thus, using public social media, designers are enabled to recombine and refine existing internal knowledge resources generated through using enterprise social media, and face-to-face interaction with co-workers, to create a new knowledge base that is useful to develop novel content of the product design. For instance, comments from the customers on a social commerce site e.g., Taobao may help designers to consider reviewing product design according to the needs of the customers. Thus, we propose that public social media usage helps designers to acquire and integrate their knowledge and ideas to create NFPD.

Secondly, the use of public social media can support EFPD through enhanced interaction with potential customers and end-users. Public social media increases the designer's interaction with potential users of the product, which enables a designer to resolve design issues in close interaction with customers, which helps improve the efficiency of the product design. In this regard, public social media platforms facilitate timely and accurate information and feedback of the customers for the product designers, to understand each other, which reduces design uncertainty for both designers and consumers, thus enabling EFPD. In summary, we hypothesize that

H3: Public social media use is positively associated with novelty-focused product design.
H4: Public social media use is positively associated with efficiency-focused product design.

### Product Design and Designer Performance

A product design is a blueprint for the value creation of market opportunities through the design of a product that is innovative and efficient to satisfy customer needs. Previous studies have focused on two main themes of product design which are NFPD and EFPD (Gerwin and Barrowman, 2002; Micheli and Gemser, 2016). NFPD focuses on designing a product that is creative and useful for the end-user, whereas, an EFPD focuses on efficient product design that is cost-effective for organization, as well as provide durable product with low cost to the customers. Two of the product design themes are not mutually exclusive, yet, depending on the organization and customer preferences, both may have a unique contribution toward customer satisfaction and eventually designer performance. Thus, focus on a particular design them is critical for the organization as it enables an organization to attract customers and ensures customer retention that is key in the current global business scenario.

Product and service design is an important factor for enhancing customer satisfaction and loyalty (Pereira et al., 2019), which define the performance of a product designer at the micro-level (Nazari-Shirkouhi and Keramati, 2017) and organizational performance at a macro level (Micheli and Gemser, 2016). Especially, NFPD can create new business opportunities, and give increase designer scope to suggest products with unique design features, which positively affects organizational performance. Firstly, NFPD enables designers to create value for the customers by providing them novel features and designs (Moon et al., 2015). A novel product creates social value for the customers and thus increases their satisfaction with the product while increasing their loyalty to the organization. In this way, designers can create the first-mover advantage for the organization and increase customer value. In addition, through NFPD, a designer may also enable organizations to satisfy customers by providing them with a novel product that is not easily acquired from other organizations. Thus, the NFPD focus of a designer helps organizations increase customer satisfaction through offering novel products that are not useful and unique from other products of competitive organizations. Together, opportunities created by the designers focus on the NFPD is expected to create value for the organization by creating a pool of satisfied and loyal customer, which also explain contributions of the designer and hence his performance.

EFPD on the other hand can also enhance the value and performance of a designer for the organization by enabling him to create a product that is lower in cost while maintaining the quality and features. EFPD encourages increased information flow among different departments of the organization and simplified designing and manufacturing processes so that the cost of the product is reduced. Therefore, designers can develop product designs that are coordinated with manufacturing, finance, and sales and marking departments to minimize the cost of the product for the customers while maintaining features and quality, which leads to increase customer satisfaction. Simultaneously, EFPD may also encourage the organization to outsource some of the production processes to decrease production costs. Thus, EFPD may increase customer satisfaction due to the low cost and high quality, and improved features of the product. Based on the above arguments, in summary, we propose that NFPD and EFPD both have unique contributions toward creating a satisfied pool of customers and help organizations to attract and retain loyal customers. Accordingly, this study proposes that

H5: Novelty-focused product design is positively associated with designer performance.
H6: Efficiency-focused product design is positively associated with designer performance.

## Methods

### Participants and Procedures

The data used in this study for empirical analysis was collected through longitudinal surveys in major cities of Pakistan. The sampling companies have full-time employees for product design, and research and development. We contacted the managing directors and HR managers of identified target companies. We explained to them the details of our research and requested their voluntary participation in our survey. After the approval from the management of the 16 companies, we requested HR managers to help us distribute questionnaires to the employees.

In phase 1, questionnaires were distributed among employees engaged in designing the products. They were asked to report demographic information (i.e., age, gender, education, experience, experience with social media, social media usage frequency), enterprise social media use, public social media use, NFPD, and EFPD. After a month of the initial survey, direct managers of the respondents were requested to report the performance of the employees.

A total of 419 employees from 16 companies were invited to complete the surveys. A total of 124 employees did not respond or provided an incomplete response, leaving a total of 295 responses (70% response rate) used as the final sample to test our model. Additionally, in phase 2, 85 managers rated the performance of the respondents. All the participants completed the survey voluntarily. Among the respondents, 158 are male (53.6%), with most of the respondents' age was <35 years (81.4%); most of the respondents have at least a bachelor's degree (94%). Table 1 shows the respondents' demographic characteristics.

**Table 1:** Demographic information of respondents.

**Measure**	**Items**	**Frequency**	**Percent**	**Measure**	**Items**	**Frequency**	**Percent**
Gender	Male	158	53.6	Age range	18–25	138	46.8
	Female	137	46.4		26–35	102	34.6
Education Level	Intermediate	18	6.1		36–45	49	16.6
	Bachelor	142	48.1		46 or above	6	2.0
	Masters or above	135	45.8	Social media usage frequency	<10	39	13.2
Social media use experience	<5 years	11	3.7		11–20	103	34.9
	5–10 years	130	44.1		21–30	106	35.9
	Over 10 years	154	52.2		31 and above	47	15.9

### Survey Items

This study followed previous research (Ali et al., 2019b; Khan et al., 2019; Bahadur and Ali, 2021) and used multi-item scales extracted from prior studies on social media and product development. We adopted the scale from Ou and Davison (2011) to measure enterprise social media using six items. This measure is extensively used in previous studies on social media (Cai et al., 2018; Pitafi et al., 2018; Wei et al., 2020). To measure the use of public social media, five items scale was adapted from van Zoonen et al. (2016). This measure is validated in other studies on social media (Van Zoonen et al., 2017). This measure assesses how people use public social media accounts for social and professional interactions, communication, and information sharing with coworkers and the public. Individual employees' public social media accounts, as well as company official pages on public social media (e.g., a company Facebook page), are used to facilitate social and work-related engagement and information exchange. Furthermore, this measure focused on Facebook, Twitter, and LinkedIn because these social media channels are predominantly used in Pakistan. A measure of 3 items to measure EFPD was adapted from Gerwin and Barrowman (2002). This is a validated measure by Naveh (2005). We adapted 3 items to measure NFPD from the study on Micheli and Gemser (2016). Each manager was asked to rate the performance of their subordinates on a 4 items scale adopted from Thompson (2005).

Previous studies have identified a number of factors that may also affect the results of our study. Therefore, in our analysis, we controlled the effect of age, gender, education, experience with social media, and social media usage frequency of each participant.

### Common Method Bias

The data for empirical analysis was collected from multiple sources at two points in time. Yet, most of the variables were self-reported collected at a single point of time, which creates the possibility of the potential effect of common method bias (Podsakoff et al., 2003). First, we performed Harman's single factor test to check this potential effect of common method bias (Harman, 1976). Analysis results reveal that 17 constructs generated eigenvalues value >1.0, accounting for 71.27% of the variance. Further, results show that the first construct with an eigenvalue of 25.32% shows that it does not account for the majority of the variance. Next, we conducted two confirmatory factor analyses on employee-rated data to compare model fit results of the four factors model with a single-factor model. The findings of confirmatory factor analyses indicate that five factors model generate reasonable fit to the data (χ^2^ = 192.04, d.f. = 113, RMSEA = 0.05, CFI = 0.89, IFI = 0.98, NFI = 0.94, and GFI = 0.93) than single-factor model (χ^2^ = 1397.86, d.f. = 119, RMSEA = 0.19, CFI = 0.61, IFI = 0.61, NFI = 0.59, and GFI = 0.57). The results of confirmatory factor analyses further indicate that it is unlikely that common method bias will affect the findings of this study.

## Results

### Analysis of Measurement Model

The validity of the scales was determined using confirmatory factor analysis (CFA). The CFA results suggested that the measurement model and dataset were well-fitted (χ^2^ = 278.28, d.f. = 179, RMSEA = 0.04, CFI = 0.97, IFI = 0.97, NFI = 0.93, and GFI = 0.92). The results suggest that all item loadings were above the recommended benchmark of 0.60 (Hair et al., 2020). We tested the convergent validity using Cronbach's alpha, composite reliability of constructs, and average variance extracted (AVE) (Fornell and Larcker, 1981; Flynn et al., 1990; Nunnally and Bernstein, 1994). Table 2 reveals that the value range of 0.83–0.97 for Cronbach's alphas was reasonably above the suggested benchmark of 0.70. The results also indicate the range of 0.81–0.97 for composite reliability were acceptable as all values are above the suggested threshold value of ranged from 0.81 to 0.97 and were above the benchmark value of 0.70. Similarly, the threshold value of AVE is 0.50. The findings of the analysis indicate a range of 0.50–0.86 for AVE, which is above the acceptable value. Together, the results confirm the convergent validity of the model. To check the discriminant validity of the measurement model, we calculated the square roots of the AVEs. As presented in Table 3, results suggest that square roots of AVEs are greater than the correlations among variables. These results suggest that discriminant validity was achieved in our measurement model. Moreover, cross-loadings of the items are presented in Table 4. The results indicate that none of the items loaded on the other constructs at with unacceptably high. Thus, the lower cross-loadings of the items further suggest a good level of discriminant validity of the measurement model. The above results suggest that the measurement model satisfies the recommended levels of convergent validity, discriminant validity, and reliability.

**Table 2:** Results of confirmatory factor analysis.

**Variable**	**Cronbach's alpha**	**Composite reliability**	**Average Variance Extracted (AVE)**
ESM	0.85	0.85	0.50
PSM	0.97	0.97	0.86
NFPD	0.80	0.81	0.59
EFPD	0.83	0.83	0.62
DP	0.84	0.84	0.56

*ESM, Enterprise social media; PSM, Public social media; NFPD, Novelty-focused product design; EFPD, Efficiency-focused product design; DP, Designer performance*.

**Table 3:** Discriminant validity.

**Variable**	**Mean**	**Std. Deviation**	**Gender**	**Age**	**Education**	**SM-UE**	**SM-UF**	**ESM**	**PSM**	**NFPD**	**EFPD**	**DP**
Gender	1.46	0.50	NA									
Age	1.74	0.81	−0.09	NA								
Education	3.40	0.60	0.04	0.07	NA							
SM-UE	2.48	0.57	−0.09	0.00	0.00	NA						
SM-UF	2.55	0.91	0.01	0.00	−0.07	−0.01	NA					
ESM	3.64	0.67	−0.06	0.02	−0.02	0.14^*^	−0.02	**0.70**				
PSM	4.41	1.03	−0.06	−0.07	0.07	0.17^**^	0.01	0.18^**^	**0.93**			
NFPD	3.23	1.03	0.00	−0.04	0.01	0.18^**^	−0.07	0.17^**^	0.19^**^	**0.77**		
EFPD	3.81	0.70	−0.03	−0.05	0.02	0.01	0.06	0.27^**^	0.14^*^	0.12^*^	**0.79**	
EP	3.39	1.01	0.00	−0.04	−0.11	0.10	0.06	0.05	0.09	0.19^**^	0.21^**^	**0.75**

*SM-UE, Social media usage experience; SM-UF, Social media usage frequency; ESM, Enterprise social media; PSM, Public social media; NFPD, Novelty-focused product design; EFPD, Efficiency-focused product design; DP, Designer performance*;

* *p < 0.05*,

** *p < 0.01. The diagonal elements are the square roots of the AVEs are in bold*.

**Table 4:** Factor loadings of the research constructs.

**Variable**	**Items**	**Factor**				
		**1**	**2**	**3**	**4**	**5**
ESM	ESM1	−0.033	**0.727**	0.055	0.025	−0.029
	ESM2	0.048	**0.723**	−0.003	−0.034	−0.004
	ESM3	0.023	**0.630**	0.001	0.050	−0.022
	ESM4	0.057	**0.670**	−0.010	−0.020	−0.016
	ESM5	−0.074	**0.696**	−0.046	0.006	−0.032
	ESM6	−0.001	**0.769**	−0.003	−0.007	0.086
PSM	PSM1	**0.878**	0.014	0.020	0.000	0.045
	PSM2	**0.958**	−0.008	−0.012	−0.002	−0.015
	PSM3	**0.968**	0.008	0.016	−0.023	−0.040
	PSM4	**0.919**	0.015	−0.028	0.024	0.017
	PSM5	**0.903**	−0.015	0.006	0.001	−0.016
NFPD	NFPD1	0.055	−0.026	−0.013	−0.011	**0.886**
	NFPD2	−0.028	−0.047	−0.057	0.040	**0.635**
	NFPD3	−0.040	0.059	0.046	−0.032	**0.774**
EFPD	EFPD1	0.063	−0.051	−0.001	**0.810**	0.027
	EFPD2	0.002	0.012	−0.011	**0.827**	−0.002
	EFPD3	−0.068	0.059	0.003	**0.720**	−0.024
DP	DP1	−0.017	0.002	**0.732**	−0.057	−0.014
	DP2	−0.003	0.033	**0.727**	−0.020	−0.053
	DP3	0.030	−0.039	**0.787**	0.013	−0.050

*Extraction Method: Maximum Likelihood, a. Rotation converged in five iterations, ESM, Enterprise social media; PSM, Public social media; NFPD, Novelty-focused product design; EFPD, Efficiency-focused product design; DP, Designer performance. Bold values indicate the factor loading of each construct*.

### Analysis of Structural Model

We used the structural equation model in AMOS 23.0 to test the structural model to analyze hypothesized relationships. Initially, fit indices indicate that our structural model reasonably fit with the data (χ^2^ = 404.75, d.f. = 292, RMSEA = 0.04, CFI = 0.97, IFI = 0.97, NFI = 0.90, and GFI = 0.91). The structural model analysis results are presented in Table 5. Specifically, the results indicate that enterprise social media usage is positively related to NFPD (β = 0.34, *p* < 0.01), and EFPD (β = 0.36, *p* < 0.001). These results show that H1 and H2 are supported by the empirical analysis. Results in Table 5 further reveal that public social media usage is significantly related to NFPD (β = 0.22, *p* < 0.01), but not with EFPD (β = 0.07, *p* > 0.05). Thus, the results indicate that H3 was accepted, but H4 was rejected at this stage. Table 5 shows that NFPD was positively correlated with designer performance (β = 0.15, *p* < 0.01: supporting H5). Results further indicate that EFPD has a significant positive effect on designer performance (β = 0.27, *p* < 0.001). Consequently, H6 was supported. Additionally, among the five control variables (age, gender, education, social media usage experience, social media usage frequency), education is found to have a significant negative effect on designer performance.

**Table 5:** Results of the structural model.

**Hypothesized relationships**	**Unstandardized coefficient**	**S.E**.	**C.R**.	**Conclusion**
H1: ESM→NFPD	0.34^**^	0.13	2.60	Supported
H2: ESM→EFPD	0.36^***^	0.08	4.20	Supported
H3: PSM→NFPD	0.22^**^	0.07	3.03	Supported
H4: PSM→EFPD	0.07	0.04	1.57	Not -supported
H5: NFPD→DP	0.15^**^	0.05	2.98	Supported
H6: EFPD→DP	0.27^***^	0.08	3.37	Supported
**Control variables:**				
Gender→DP	0.02	0.10	0.19	
Age→DP	−0.01	0.06	−0.23	
Education→DP	−0.16^*^	0.08	−2.00	
SM-UE→DP	0.10	0.09	1.15	
SM-UF→DP	0.05	0.05	0.97	

*ESM, Enterprise social media; PSM, Public social media; NFPD, Novelty-focused product design; EFPD, Efficiency-focused product design; DP, Designer performance; SM-UE, Social media usage experience; SM-UF, Social media usage frequency*;

* *p < 0.05*,

** *p < 0.01*,

*** *p < 0.001*.

## Discussion and Contributions

### Discussion

The purpose of this study was to investigate the relationship between social media use, product design, and designer performance. Based on OL theory and insights from information systems and product design literature, this study investigated the impact of enterprise social media and public social media by designers on product design and ultimately on designer performance. The empirical analysis of this study generated results, which are in line with previous studies and provide novel insights into the phenomena. Particularly, the findings empirically suggest that the role of NFPD and EFPD have interesting implications to explain the role of product design focus and designer performance. In addition, the results of the analyses show that NFPD and EFPD are significantly affected by the use of enterprise social media. Furthermore, results show that public social media use is more related to NFPD. These findings contribute to the existing literature on social media use and product design (D'Andrea et al., 2015).

The findings of the empirical analysis reveal that public social media use is not associated with EFPD. One possible reason for this insignificant relationship might be that in public social media, designers look for opinions and views about product design more than on the features and quality of the product. In this line, the literature on social media explains that the use of social media for interaction, communication, and knowledge exchange enables creativity and innovation of the employees (Ali et al., 2019a, 2020a). In other words, the use of public social media does not facilitate the requirements of EFPD development.

The results also disclose the impact of NFPD and EFPD on designer performance. Specifically, results indicate that EFPD is strongly related to designer performance. Besides, results also support the NFPD has a significant impact on customer satisfaction. The results indicate the both NFPD and EFPD are important factors in describing customer satisfaction.

### Theoretical Contributions

Our research has important contributions to the theory. First, this study contributes to social media literature by theoretically developing a model and empirically testing the direct relationship between social media use and product design. Though, research has revealed the implications of social media for communication, knowledge sharing, learning, creativity, and innovation (Davison et al., 2018; Gong et al., 2019; Ali et al., 2020b; Laitinen and Sivunen, 2020). However, not theoretically nor empirically, it is clear whether social media is also effective for product design. This current research extends the literature on social media by explaining the impact of social media on product design.

Second, this study extends the literature on social media and product design by exploring how different social media platforms impact product design focuses. Specifically, this study investigates how enterprise social media and public social media are linked with NFPD and EFPD development. Thus, this investigation highlights the importance of using enterprise social media and public social media to facilitate product designing. This study, in line with Meuser et al. (2016), thus, indicates that integrated use of both enterprise social media and public social media has numerous benefits for the organizations. The results interestingly highlight that public social media use does not significantly affect EFPD. However, both kinds of social media platforms have a significant impact on NFPD and EFPD.

Third, based on OL theory (Real et al., 2006), this study contributes to the existing social media and product design literature by highlighting the importance of using different social media tools to design products that can satisfy the novelty and efficiency requirements of the customers, hence leading to designer performance. Thus, we expand the literature on organizational learning and social processes of innovation by engaging different stakeholders in the product design process using social media platforms. This study indicates that a full understanding of the complex and joint nature of social media platforms, design focus may bring novel insights for the organizations to attract and retain satisfied customers and improved designer performance.

### Managerial Implications

Our research has important implications for managers. First, designers must pay attention to their product design. NFPD and EFPD can create value for both organization and the customers. Therefore, managers who need to improve organizational performance need to think about whether the novelty of a product design or efficiency of a product design will create value for them. Second, managers should also be aware of the important role social media plays in enabling designers to get insight, ideas, and information from different sources such as co-workers and potential users. Specifically, the empirical findings call for a strategic integration between enterprise social media use and public social media use. Designers who are facilitated to simultaneously use enterprise social media and public social media can create novel and efficient designs according to the organizational structural capabilities settings while satisfying customer requirements. It can potentially include the media rich environments i.e., virtual or augmented reality to communicate in effective manner. Thus, we propose that managers should adjust the balance of using enterprise social media and public social media usage by the employees to achieve the required NFPD and EFPD.

### Limitations and Future Directions

Despite the contributions to the theory and practice, we note this study has the following limitations, which indicate research directions for future scholars. The survey for this study was conducted in Pakistan. Pakistan is a developing country and has its own political, economic, and cultural dimensions. Although, such differences are unavoidable in research, however, these may limit the generalizability of our findings. Thus, we encourage future studies to test our model in their own socio-cultural context of their country. Second, in this study, we measured designer performance using subjective measures rather than using an objective measures. Future scholars are thus suggested to use objective measures such as product sales. Third, our model investigates enterprise social media use and public social media use as two types of social media used by the employees. Yet, employees use enterprise social media and public social media for work-related purposes and also for social-related purposes. Research has demonstrated that different usage purposes have unique implications for the performance of the employee (Ali-Hassan et al., 2015; Ali et al., 2019a; Cao and Yu, 2019). Thus, we recommend future studies to investigate the impact of work-related social media use and social-related social media use on the NFPD and EFPD.

## Data Availability Statement

The raw data supporting the conclusions of this article will be made available by the authors, without undue reservation.

## Author Contributions

NH contributed to conception of this study. MH performed the statistical analysis. MG, FA, and NH wrote sections of the manuscript. All authors contributed to manuscript revising, read, and approved the submitted version.

## Conflict of Interest

FA was affiliated with the company Anhui Xinhua Media Co., Ltd. The remaining authors declare that the research was conducted in the absence of any commercial or financial relationships that could be construed as a potential conflict of interest.

## Publisher's Note

All claims expressed in this article are solely those of the authors and do not necessarily represent those of their affiliated organizations, or those of the publisher, the editors and the reviewers. Any product that may be evaluated in this article, or claim that may be made by its manufacturer, is not guaranteed or endorsed by the publisher.
